# Endoglin integrates BMP and Wnt signalling to induce haematopoiesis through JDP2

**DOI:** 10.1038/ncomms13101

**Published:** 2016-10-07

**Authors:** June Baik, Alessandro Magli, Naoyuki Tahara, Scott A. Swanson, Naoko Koyano-Nakagawa, Luciene Borges, Ron Stewart, Daniel J. Garry, Yasuhiko Kawakami, James A. Thomson, Rita C. R. Perlingeiro

**Affiliations:** 1 Department of Medicine, Lillehei Heart Institute, University of Minnesota, Minneapolis, Minnesota 55455, USA; 2 Department of Genetics, Cell Biology, and Development, University of Minnesota, Minneapolis, Minnesota 55455, USA; 3 Stem Cell Institute, University of Minnesota, Minneapolis, Minnesota 55455, USA; 4 Regerative Biology, Morgridge Institute for Research, Madison, Wisconsin 53715, USA

## Abstract

Mechanisms of haematopoietic and cardiac patterning remain poorly understood. Here we show that the BMP and Wnt signalling pathways are integrated in an endoglin (Eng)-dependent manner in cardiac and haematopoietic lineage specification. Eng is expressed in early mesoderm and marks both haematopoietic and cardiac progenitors. In the absence of Eng, yolk sacs inappropriately express the cardiac marker, Nkx2.5. Conversely, high levels of Eng *in vitro* and *in vivo* increase haematopoiesis and inhibit cardiogenesis. Levels of Eng determine the activation of both BMP and Wnt pathways, which are integrated downstream of Eng by phosphorylation of Smad1 by Gsk3. By interrogating Eng-dependent Wnt-mediated transcriptional changes, we identify *Jdp2* as a key Eng-dependent Wnt target, sufficient to establish haematopoietic fate in early mesoderm when BMP and Wnt crosstalk is disturbed. These studies provide mechanistic insight into the integration of BMP and Wnt signalling in the establishment of haematopoietic and cardiac progenitors during embryogenesis.

## Abstract (web summary)

How both BMP and Wnt signalling pathways regulate lineage specification early in development is unclear. Here, the authors show that endoglin via *Jdp2*, an AP-1 family member, modulates BMP and Wnt signalling to commit mesodermal progenitors to a haematopoietic fate at the expense of the cardiac lineage.

## Graphical abstract

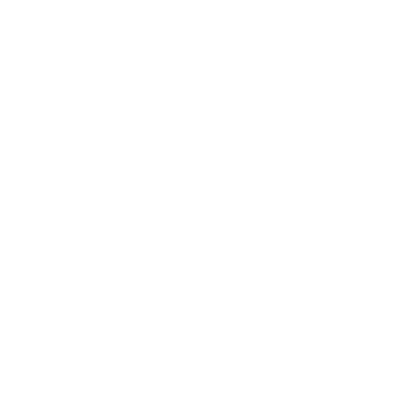

In the developing embryos, haematopoietic and cardiac cells arise as subpopulations of lateral plate mesoderm^1^,^2^, but the phenotypic profiles of these progenitors as well as the molecular mechanisms regulating their specification remain not well defined. It is known that BMP and Wnt signals from the extra-embryonic region and epiblast, respectively, are required for mesoderm induction^3^. Homozygous deletion of BMP4 and its type 1 receptor BMPR1a, as well as Wnt3 and its intercellular effector β-catenin, result in embryonic lethality due to the failure of mesoderm formation and subsequent patterning. Wnt signals along with BMP are also critical to establish the prospective cardiac population^1^ by directing the migration of cardiac progenitors from the primitive streak to the heart forming region^4^. This convergence of Wnt and BMP signalling is also observed during haematopoiesis. Studies involving avian embryo manipulation and *in vitro* differentiation of murine embryonic stem (ES) cells have demonstrated that the combination of Wnt and BMP signalling is essential for haematopoietic specification and commitment towards the primitive erythroid lineage^1^,^5^,^6^.

We previously reported that endoglin (Eng), an ancillary receptor for the transforming growth factor-β superfamily, is required for early haematopoietic lineage specification, and that Eng acts by modulating output of the BMP signalling pathway^7^,^8^,^9^. Haematopoiesis was found impaired in both Eng-deficient differentiating ES cells^10^ and embryos^9^. On the other hand, upregulation of Eng using doxycycline-inducible Eng (iEng) ES cells resulted in enhanced haematopoietic differentiation and suppressed cardiac differentiation^7^, suggesting a key role for this receptor in early cell fate specification.

Using zebrafish and mouse models, in the present study, we provide *in vivo* evidence for the dual role of Eng in blood and cardiac lineage specification, and show that this effect results from enhanced Wnt activity, which is directly regulated by Eng. In fact, levels of Eng determine the activation potential of both BMP and Wnt pathways, and phosphorylation of Smad1 by Gsk3 is the point of integration between BMP and Wnt signals. We further show that Eng identifies early progenitors endowed with haematopoietic and cardiac potential that are Eng^+^Flk-1^+^ and Eng^+^Flk-1^−^, respectively, demonstrating that Eng marks not only haematopoietic but also cardiac cell fate within the mesoderm population. Through whole transcriptome analyses of these early haematopoietic and cardiac progenitor fractions, in the presence or absence of the Wnt inhibitor IWR, we identified *Jdp2*, a member of the AP-1 transcription factor family, as a key Eng-dependent downstream target of Wnt signalling. Importantly, we show *Jdp2* expression is sufficient to establish haematopoietic fate in early mesoderm when BMP and Wnt crosstalk is disturbed, providing mechanistic insight into the establishment of haematopoietic and cardiac progenitors during embryogenesis.

## Results

### Effect of Eng induction *in vitro* and *in vivo*

**Figure 1:**
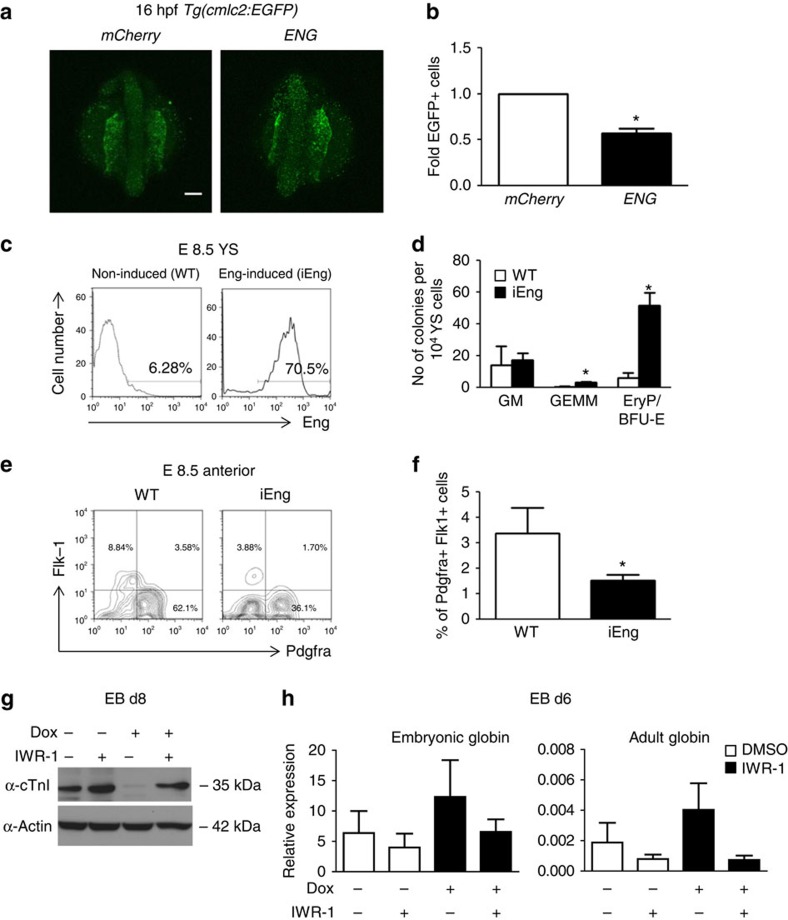
The dual effect of endoglin in early cell specification requires Wnt activation. (**a**,**b**) *ENDOGLIN (ENG)* and control *mCherry* mRNAs were injected into *Tg(cmlc2:EGFP)* zebrafish embryos. (**a**) Embryos were imaged to visualize cardiac progenitors (EGFP^+^ cells) at 16 h.p.f. All views are dorsal. Scale bar, 100 μm. (**b**) Graph bars show the fold difference of EGFP^+^ cells, quantified by FACS, between *ENG*- and *mCherry*-injected embryos, which show reduced frequency of cardiac progenitors upon ENG induction. Bars represent s.e.m. from three independent experiments. *P<0.05 by *t*-test. (**c**–**f**) YSs and anterior regions of E8.5 endoglin-induced mouse embryos were collected and analysed for haematopoietic and cardiac potential, respectively. (**c**) FACS analyses show induction of endoglin expression in the yolk sac of E8.5 iEng mice (right panel). (**d**) YSs from Eng-induced and control (WT) counterparts were assessed for their haematopoietic activity. Significant increased numbers of erythroid and GEMM colonies were observed upon endoglin induction. Error bars indicate s.e.m. from two litters of E8.5 Eng-induced (*n*=13) and WT littermate control (*n*=4) YSs. *P<0.05 by *t*-test. (**e**) Representative FACS profiles for the expression of Pdgfra and Flk-1 in the anterior regions of E8.5 endoglin-induced mouse embryos and control (WT) counterparts. (**f**) Quantification of Pdgfra^+^Flk-1^+^ cells confirms cardiac suppression upon induction of endoglin. Bars indicate s.e.'s from two litters of E8.5 Eng-induced (*n*=13) and WT littermate control (*n*=4) embryos. *P<0.05 by *t*-test. (**g**,**h**) iEng ES cells were differentiated as EBs. Dox was added to EB cultures from day 2, and IWR-1 from day 4 to 6. EB cultures were analysed for cardiac and haematopoietic differentiation at days 8 and 6, respectively. (**g**) Western blot analyses reveal rescue of cTnI expression in Eng-induced EBs that had been treated with IWR-1. (**h**) Gene expression analysis for embryonic and adult globins demonstrates that IWR-1 counteracts the stimulatory effect of endoglin in erythropoiesis. Transcript levels are normalized to *Gapdh*. Bars indicate s.e.'s from three independent experiments.

Our previous findings using *in vitro* differentiation of iEng ES cells suggest a time-dependent effect, as Eng overexpression from day 2 to 8 of embryoid body (EB) differentiation repressed cardiogenesis, whereas Eng overexpression from day 4 to 8 had no effect^7^. To better understand this temporal dependence, we performed a fine time-course analysis, which revealed a short window from day 2 to 3 in which overexpression of Eng enhances haematopoiesis and represses cardiac differentiation (Supplementary Fig. 1a,b). This is the time at which mesoderm is being specified to haematopoietic or cardiac fate. To investigate whether Eng induction results in cardiac suppression *in vivo*, we collected E8.5 mouse embryos, a time point with abundant cardiovascular progenitors^11^, and transduced these with lentiviral vectors encoding Eng or empty vector (control pSAM; Supplementary Fig. 1c). Control explant cultures gave rise to large clusters of cells positive for cTnI (Supplementary Fig. 1d, lower left), in contrast to Eng-transduced explants, which presented significantly reduced numbers of cells expressing cTnI (Supplementary Fig. 1d, lower right). In a complementary approach, we utilized a cardiac reporter transgenic zebrafish, in which GFP identifies cardiac progenitors that are positive for myosin light chain 2 *Tg*(*cmlc2:EGFP*)^12^, to determine whether Eng induction disturbs cardiac specification *in vivo*. For this, *Tg(cmlc2:EGFP)* embryos at the 1–2-cell stage were injected with human *ENG* messenger RNA (mRNA), which has been previously demonstrated to cross-react in zebrafish^13^, or with control *mCherry* mRNA. Consistent with our *in vitro* and *ex vivo* studies, live imaging analyses at 16 h post fertilization (h.p.f.) demonstrated impaired cardiogenesis in *ENG*-injected embryos, evidenced by reduced levels of GFP expression (Fig. 1a, right panel) when compared with *mCherry*-injected controls (Fig. 1a, left panel). Fluorescence-activated cell sorting (FACS) analyses confirmed that the number of EGFP^+^ (cmlc2^*+*^) cells within the ENG^*+*^ population was reduced when compared with those within the control mCherry^+^ population (Fig. 1b), further indicating that enhanced expression of Eng represses cardiac specification during gastrulation

To further evaluate the activity of Eng in cardiac versus haematopoietic specification, we utilized a potent hemogenic tissue, E9.5 yolk sac (YS). *Eng*^−/−^ E9.5 YSs have been reported to exhibit severely reduced haematopoietic colony activity as well as marked downregulation of *Scl*, *Gata1*, *Gata2* and globins^9^. Thus, we interrogated here whether the role of Eng was to actively repress other lineages in E9.5 YSs (Supplementary Fig. 1e). At the gene expression level, we observed significant upregulation of *Nkx2.5* (Supplementary Fig. 1f, left), one of the earliest markers of cardiac development^14^. Expression levels of *Gata4* and *cTnI* also trended towards elevated levels in *Eng*-null YSs (Supplementary Fig. 1f). These data suggest a role for Eng in enabling the haematopoietic program by preventing the expression of cardiac-specific genes at hemogenic sites during mesoderm specification.

We then evaluated the effect of overexpressing Eng *in vivo* during mouse embryo development using iEng mice, which we generated from the previously described iEng ES cells^7^. As observed in Fig. 1c, robust induction of Eng was observed in E8.5 YSs from embryos whose mothers were treated with dox during pregnancy. Next, equal numbers of YS cells from these embryos were directly plated into methylcellulose containing haematopoietic cytokines to examine haematopoietic colony activity. As observed in Fig. 1d, Eng-induced YSs gave rise to increased numbers of haematopoietic colonies when compared with non-induced YSs, confirming that Eng induction results in enhanced haematopoiesis. To assess cardiogenesis, we dissected E8.5 embryos, and separated anterior (containing cardiac progenitors) from posterior domains (used for genotype). Expression of Pdgfra and Flk-1 identifies cardiac progenitors at this developmental stage^15^,^16^, therefore we analysed the expression of these markers in the Eng^+^ cell fraction of the anterior regions of these embryos. In agreement with our hypothesis, the frequency of Pdgfra^+^Flk-1^+^ cells within the Eng^+^ population was found significantly decreased when Eng was induced (Fig. 1e,f). Taken together, these *in vivo* data confirm dual positive/negative functions for Eng in the establishment of haematopoietic and cardiac progenitors, respectively, during mesoderm specification.

### Eng directly regulates Wnt signalling

Based on the critical role of Wnt signalling during early haematopoiesis and cardiogenesis^1^,^6^,^17^, we investigated whether Wnt activation is involved in Eng-mediated mesoderm specification. For this, we assessed the effect of the canonical Wnt signalling inhibitor, IWR-1, in Eng-induced EB cultures, and found that the inhibitory effect of Eng induction (+dox) on cardiac differentiation was abrogated by inhibition of the canonical Wnt signalling pathway (Fig. 1g). To determine whether this would also be the case *in vivo*, we cultured Eng- and mock-transduced embryo explants in the presence or absence of IWR-1. In agreement with the *in vitro* EB data, addition of IWR-1 to Eng-transduced primary cultures counteracted the inhibitory effect of Eng on cardiac differentiation (Supplementary Fig. 1g,h). We also observed that Wnt inhibition by IWR-1 in EB cultures negatively affected the ability of Eng to induce haematopoiesis (Fig. 1h), demonstrating that the function of Eng at this early stage of development requires active Wnt signalling.

**Figure 2:**
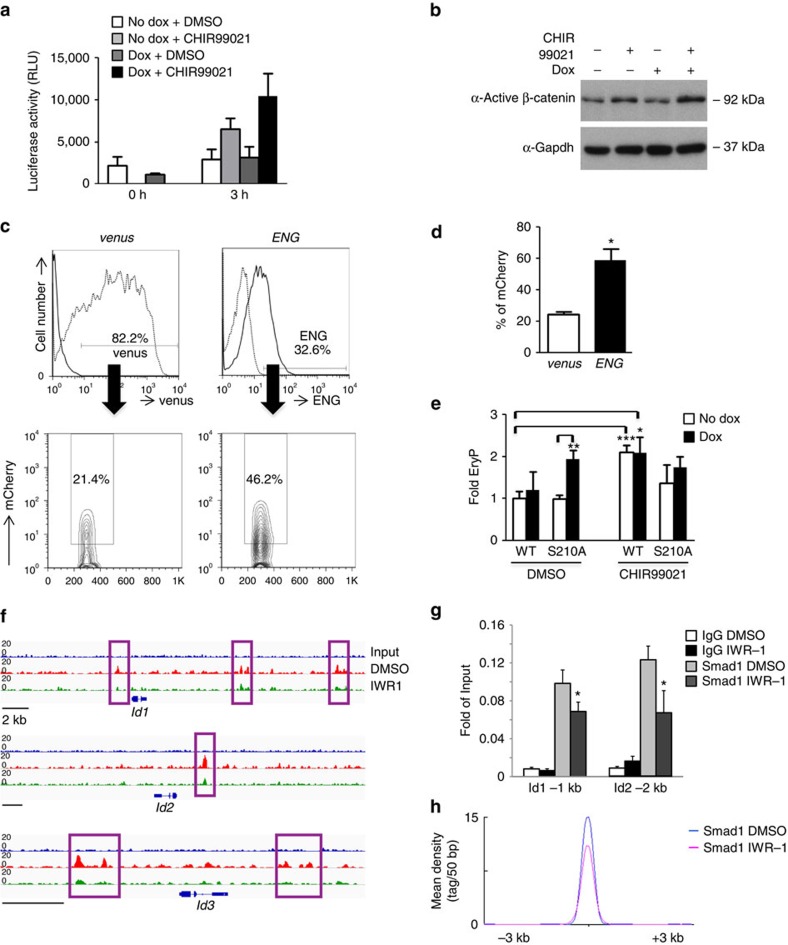
Endoglin-mediated Wnt activation enhances Smad1 activity to promote haematopoiesis. (**a**,**b**) A β-catenin-dependent reporter (7TFC) was introduced into the iEng ES cell line (7TFC:iEng). 7TFC:iEng ES cells were differentiated as EBs, and dox was added to the culture medium from day 2. The GSK3β-inhibitor CHIR99021 was added to cultures 24 h later, and samples were collected at 0 and 3 h. (**a**) Luciferase activity denotes Wnt stimulation in these cultures, and peak is observed after 3 h solely in endoglin-induced EB samples that had been exposed to CHIR99021. Graph is representative of three independent experiments. (**b**) Respective western blot analyses for active β-catenin in EB cultures following 3 h of Wnt stimulation. Consistently, higher levels of active β-catenin are observed in cultures that had been exposed to dox (iEng) and CHIR99021. Gapdh was used as loading control. (**c**,**d**) *ENG* and control *venus* mRNAs were injected into *Tg(7xTCF:mCherry)* zebrafish embryos. (**c**) Representative FACS profiles for mCherry expression in micro-injected embryos. mCherry expression from *venus-* and *ENG-*injected groups were gated in either venus- or ENG-positive cells, respectively. (**d**) Quantification of mCherry^+^ cells confirms enhanced Wnt activity upon ENG overexpression. Bars represent s.e.m. from three independent experiments. *P<0.05 by *t*-test. (**e**) Haematopoietic activity in D4 EBs in ES cells that had been engineered to inducibly express Smad1 carrying a mutation at the GSK3β conserved site (S210) or WT Smad1. Dox was added from day 2 to 4 of EB differentiation and CHIR99021 was added from day 3 to 4. Bars indicate s.e.'s from three independent experiments. *P<0.05, **P<0.01, ***P<0.001 by *t*-test. (**f**) Integrative Genome Viewer (IGV) tracks for *Id1*, *Id2* and *Id3* genomic regions from Smad1 ChIP-seq experiment performed on D4 EBs treated for 24 h with dimethylsulphoxide (DMSO) or IWR-1. Squares indicate Smad1 bound regions. Scale bar, 2 Kb. (**g**) Validation of Smad1 binding at the Id1 (−1 Kb) and Id2 (−2 Kb) sites by ChIP–qPCR. Graph represents the average plus s.e. from five independent experiments. IWR-1 treatment decreases Smad1 binding at these two sites. *P<0.05 by *t*-test. (**h**) Tag density distribution of ChIP-seq reads across a ±3 kb region centered on Smad1 peaks. IWR-1 treatment affects the genome wide binding of Smad1.

To determine whether Eng induction promotes Wnt activation, we generated a β-catenin-dependent reporter ES cell line. This reporter (7xTcf-FFluc//SV40-mCherry) contains 7Tcf-binding sites, allowing us to monitor Wnt activity using a luciferase reporter assay^18^. We subjected Eng-induced or control cultures to a brief pulse of Wnt signal (3 h of exposure to the GSK3β inhibitor CHIR99021), and observed that reporter activity was increased in Eng-induced cultures when compared with non-induced or mock cultures (DMSO treated; Fig. 2a). The increased reporter activity observed in the Eng-induced sample that had been treated with CHIR99021 was confirmed by western blot using an antibody specific for active β-catenin (Fig. 2b). This finding was further corroborated *in vivo* utilizing *Tg(7xTCF:mCherry)* zebrafish embryos, which at 12 h.p.f. exhibit transgenic reporter expression regulated by Wnt/β-catenin signalling, as measured by mCherry fluorescence^19^. We injected 1–2-cell stage zebrafish embryos with *ENG* mRNA or control *venus* mRNA (Fig. 2c, upper panels), and at 12 h.p.f., injected embryos were collected and analysed for mCherry expression. We observed that mCherry expression within the ENG^+^ cell fraction in *ENG*-injected embryos was significantly increased when compared with that found within the venus^+^ cell fraction in *venus-*injected controls (Fig. 2c, lower panels; Fig. 2d). As expected, mCherry expression within the ENG^−^ cell fraction of *ENG*-injected embryos was similar to control levels, ∼21% (Supplementary Fig. 2a). Moreover, equivalent results were obtained when we injected an *ENG* mutant construct encoding a deletion of the orphan domain (OD; Supplementary Fig. 2b,c), which is required for ENG expression and ligand binding^20^. Taken together, these data confirm that Eng induction enhances Wnt activity.

The stimulatory effect of Eng in haematopoiesis was previously shown to involve BMP signalling, as evidenced by the decreased frequency of haematopoietic activity correlating with reduced levels of Smad1/5/8 phosphorylation when dorsomorphin, a BMP signalling inhibitor, was added to these cultures^7^. It has been shown in *Xenopus* that Smad1, the downstream effector of BMP signalling, is also a target of Gsk3-mediated phosphorylation^21^. Gsk3 is a component of the Wnt degradation complex, and inhibition of this kinase, which maintains the Wnt pathway activate, contributes to the stabilization of activated Smad1 and ultimately strengthens BMP signalling^21^. Consistent with this, western blot analyses of day 4 Eng-induced EB cultures revealed that increased levels of phosphorylated Smad1 (S456/8 and S426/8) were accompanied by higher levels of dephosphorylated active β-catenin and inhibitory forms of Gsk3 (pGsk3 α/β S21/9) (Supplementary Fig. 2d). To determine whether Eng regulates both BMP and Wnt signals to enhance Smad1 activity and, consequently, increased haematopoietic commitment from mesoderm, we investigated whether sustained Wnt-deregulated Smad1 activity could phenocopy Eng overexpression. We generated an ES cell line with inducible expression of mutant Smad1 (S210A) lacking the Gsk3-mediated phosphorylation site (Supplementary Fig. 2e,f), which has been shown to be responsible for Smad1 degradation^21^. As hypothesized, enhanced haematopoiesis was observed only in Smad1 S210A dox-induced cultures (Fig. 2e, left), similar to the effect of Eng induction in this developmental stage^7^. Induction of wild-type (WT) Smad1 had no effect on colony formation. Importantly, whereas Wnt stimulation with CHIR resulted in enhanced haematopoiesis in induced WT Smad1 cultures (iSmad1 WT), no effect was observed in the S210A mutant (Fig. 2e, right), confirming that Wnt-mediated enhancement of haematopoiesis is dependent on the phosphorylation of Smad1 at S210.

To gain further insight into how integration of BMP and Wnt pathways regulates the commitment of mesoderm, we performed chromatin immunoprecipitation in D4 EBs followed by high-throughput sequencing (ChIP-seq) for Smad1 in the absence or presence of IWR-1. In agreement with the previous reports^22^, Smad1 binding was detected at the regulatory elements of known BMP targets *Id1*, *Id2* and *Id3* (Fig. 2f), which was also validated by ChIP–quantitative PCR (qPCR; Fig. 2g). Importantly, IWR-1 treatment negatively affected Smad1 binding at specific loci (Fig. 2f,g) and genome wide (Fig. 2h; Supplementary Fig. 2i). Among the 10,466 sites identified, Smad1 binding was impaired at ∼3,500 sites and disrupted at ∼6,500 sites (respectively, groups A and B—Supplementary Fig. 2i), thus supporting the essential role of BMP and Wnt integration to regulate Smad1 activity at the genome-wide level. Interestingly, we also observed Smad1 binding at *Tbx5* and Wnt inhibitory factor1 *(Wif1*; Supplementary Fig. 2g,h). *Tbx5*, along with *Nkx2.5*, is a key transcription factor for cardiac specification, as well evidenced by studies showing that disruption of its interaction with chromatin remodelling complex resulted in impaired cardiogenesis^23^. Considering that BMP and Wnt integration is critical for cardiac commitment, our data suggest that BMP may repress Tbx5 expression in mesodermal cells, which could account for Eng-mediated cardiac repression. Wif1 has been identified as a Smad1 target and it has been postulated to modulate BMP and Wnt activities in the developing mouse lung^24^. In agreement with Xu and colleagues, we observed that Smad1 occupies similar regions in the promoter and first intron of the *Wif1* gene (Supplementary Fig. 2g). Taken together, these data demonstrate that BMP and Wnt modulation is critical not only for Smad1 stability but also for the genome-wide occupancy of its binding sites in mesodermal cells.

### Haematopoietic and cardiac progenitors express Eng

**Figure 3:**
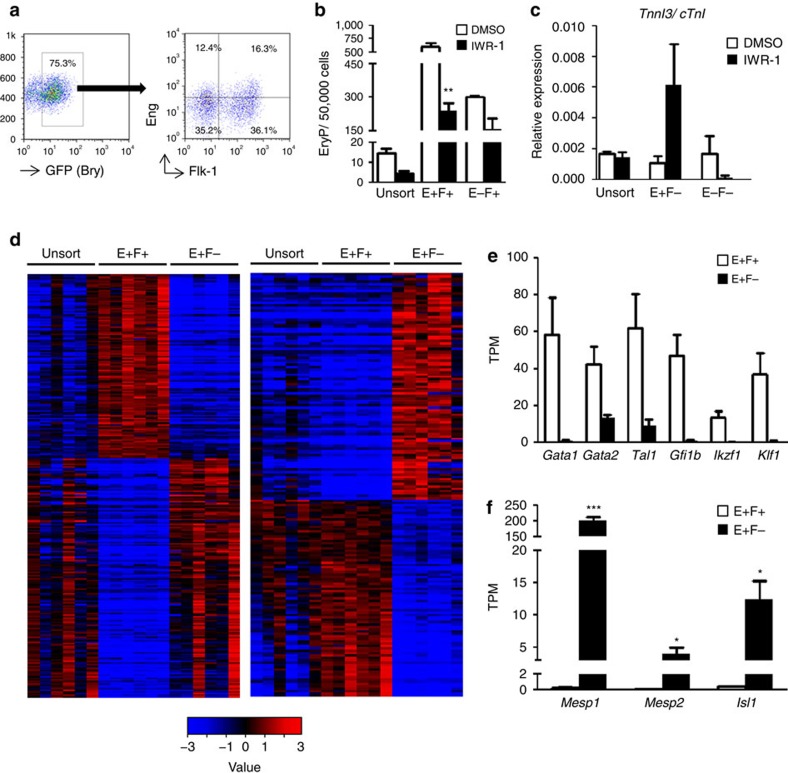
Endoglin identifies cells with both haematopoietic and cardiac potential from Bry^+^ mesoderm. (**a**) Representative FACS plots show GFP (Bry) expression in day 3 EBs (left) and subsequent analysis of gated GFP^+^ (Bry^+^) cells for the expression of Eng and Flk-1 (right). Subfractions were sorted, reaggregated for 24 h and subsequently used for analysis (**b**,**d**–**f**) or further differentiation (**c**). (**b**) Primitive erythroid colony activity in E^+^F^+^ and E^−^F^+^, and unsorted reaggregates show increased numbers of EryP colonies in the E^+^F^+^ subfraction (white bar), which is specifically abrogated by IWR-1 (black bar). Bars indicate s.e.'s from three independent experiments. **P<0.01 by *t*-test. (**c**) Cardiac potential. Reaggregates from unsorted, E^+^F^−^ and E^−^F^−^ subfractions were plated as monolayer for 5 days in the presence of IWR-1 or dimethylsulphoxide (DMSO) and analysed for the expression of *cTnni3/cTnI*. (**d**–**f**) Heatmap of RNA-Seq analysis shows genes that are distinctly expressed in either E^+^F^+^ or E^+^F^−^ (**d**). Analyses were performed with three independent biological samples. Values are log_2_(estimated expression/mean estimated expression of the row), with blue indicating negative values. (**e**) Expression levels of selected haematopoietic genes found upregulated in the E^+^F^+^ subfraction are shown as median-normalized transcripts per million (TPM). (**f**) Early cardiac transcription factors *Mesp1*, *Mesp2* and *Isl1* are selectively expressed in the E^+^F^−^ subfraction. *P<0.05; ***P<0.001 by *t*-test.

To determine whether Eng expression regulates haematopoietic and cardiac cell fate specification from uncommitted mesodermal progenitors, we utilized a GFP-Brachyury (Bry) reporter ES cell line^25^. In this cell line, GFP is knocked into the *Bry* locus, allowing us to monitor Bry expression during EB differentiation, and, importantly, to isolate early mesodermal cells. Accordingly, using this reporter ES cell line, Keller and colleagues have demonstrated that in day 3 EBs, haematopoietic progenitors are enriched within the Bry^+^Flk-1^+^ cell fraction, whereas cardiac cells are predominantly in the Bry^+^Flk-1^−^ cell subpopulation^11^,^25^. Based on this evidence, we investigated the expression of endoglin (Eng or E) in combination with Flk-1 (F) in day 3 Bry^+^ EB cells. As shown in Fig. 3a, four distinct cell subpopulations were identified: E^+^F^+^, E^+^F^−^, E^−^F^+^ and E^−^F^−^. These subfractions were FACS-purified, and sorted cells were reaggregated for 24 h in the absence or presence of IWR-1 to determine whether Wnt activity would affect Eng-mediated mesoderm specification. We examined the haematopoietic potential in reaggregates from E^+^F^+^ and E^−^F^+^ subpopulations by plating these in methylcellulose containing haematopoietic cytokines. Our results showed a significant enrichment in haematopoietic activity in the Bry^+^ cell fraction that is double positive for Eng and Flk-1 (E^+^F^+^) when compared with the unsorted group (30-fold), and remarkably also superior than the E^−^F^+^ subpopulation (2-fold) (Fig. 3b, white bars). This enrichment was abrogated by IWR-1, indicating that Wnt activity is required for the haematopoietic commitment of E^+^F^+^ cells (Fig. 3b, black bars). The inhibitory effect was not observed in cells negative for Eng (E^−^F^+^; Fig. 3b). Consistent with the previous data^25^, only very few haematopoietic colonies were observed when the Flk-1-negative cell population was assayed (E^+^F^−^ and E^−^F^−^; Supplementary Fig. 3a). To determine their cardiac potential, reaggregates from E^+^F^−^ and E^−^F^−^ populations were plated as monolayers in the presence IWR-1. Remarkably, contractile cardiomyocytes were observed only in the E^+^F^−^ group with IWR-1 (Supplementary Movies 1–3). These observations were corroborated by analyses for the cardiac functional *Tnni3* gene (Fig. 3c), which showed prominent expression in the Bry^+^E^+^F^−^ (fourfold higher than unsorted and Bry^+^E^−^F^−^). These results reveal that Eng identifies haematopoietic and cardiac progenitors within early Bry^+^ mesoderm.

To further characterize the molecular signature of these early Bry^+^E^+^F^+^ and Bry^+^E^+^F^−^ blood and cardiac subpopulations, we performed transcriptional profiling by RNA-Seq (Fig. 3d). Among the genes exclusively upregulated in the E^+^F^+^ population, we found *Gata1*, *Gata2*, *Tal1/Scl* and *Klf1* (Fig. 3e), which are known to regulate primitive erythropoiesis during embryogenesis^26^,^27^,^28^, as well as *Gfi1b* (Fig. 3e). On the other hand, the E^+^F^−^ population was characterized by high expression of *Mesp1*, *Mesp2* and *Isl1* (Fig. 3f), key regulators of early cardiac specification^29^,^30^. *Gata4* expression was also found significantly upregulated in the E^+^F^−^ population, while other cardiac-specific genes, *Nkx2.5*, *Tbx5*, *Mef2C* and *Hand2* were expressed at very low levels at this early developmental stage (Supplementary Fig. 3b). These findings are in agreement with the data obtained in the haematopoietic and cardiac assays (Fig. 3b,c; Supplementary Movies 1–3), and confirm that E^+^F^+^ and E^+^F^−^ are, respectively, endowed with haematopoietic and cardiac potential, demonstrating that Eng marks not only haematopoietic potential but also cardiac cell fate within the mesoderm population.

### Jdp2 mediates Eng's effects on haematopoiesis

**Figure 4:**
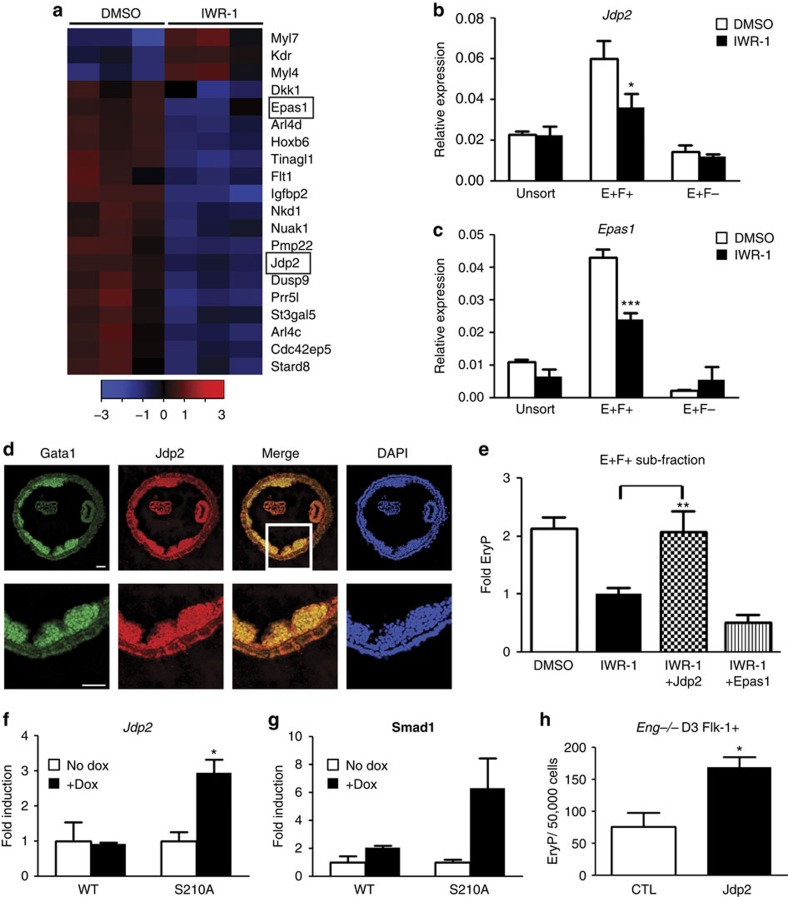
Jdp2 is a target of Eng-mediated BMP and Wnt signals to enhance haematopoietic commitment. (**a**) Heatmap of E^+^F^+^ RNA-seq from EB samples treated either with dimethylsulphoxide (DMSO) or IWR-1. Heatmap values are log_2_(estimated expression/mean estimated expression of the row), with blue indicating negative values. (**b**,**c**) Validation qPCR confirms that haematopoietic candidate genes *Jdp2* (**b**) and *Epas1* (**c**) are mostly expressed in the E^+^F^+^ fraction, and this is counteracted by IWR-1. Data represent three independent biological samples. *P<0.05; ***P<0.001 by *t*-test. (**d**) Confocal images of E7.5 YS cryosections reveal that Jdp2 is expressed in Gata1^+^ primitive erythrocytes present within the blood island. Close-up images (white box) are shown in the lower panel. 4,6-Diamidino-2-phenylindole (DAPI) stains the nuclei. Scare bars, 50 μm. (**e**) Enforced expression of Jdp2 rescues erythroid colony activity in E^+^F^+^ cells cultured in the presence of IWR-1, whereas Epas1 does not. Bars indicate s.e.'s from three independent experiments. **P<0.01 by one-way analysis of variance. (**f**,**g**) Expression levels of *Jdp2* (**f**) and *Smad1* (**g**) in day 4 EBs from inducible WT and S210A mutant Smad1 ES cells. Transcript levels are normalized to no dox group. Bar indicates s.e.'s from two independent experiments. *P<0.05 by *t*-test. (**h**) Enforced expression of Jdp2 rescues impaired primitive erythroid colony activity of Flk-1^+^ cells from *Eng*^−/−^ day 3 EBs. Bars indicate s.e.'s from two independent experiments. *P<0.05 by *t*-test.

To define how Eng-mediated BMP and Wnt modulation promotes haematopoietic commitment, we identified genes differentially expressed by IWR-1 in the E^+^F^+^ population (Fig. 4a). Among the genes showing differential expression in the control compared to IWR-1 group, we prioritized *Jdp2* and *Epas1* as candidates. These genes have been reported to be important for adult haematopoiesis^31^,^32^, and our data suggest a novel function in early cell fate specification. The expression levels of these genes were validated by qPCR, and the expression of *Jdp2* and *Epas1* was distinctively inhibited by IWR-1 in the E^+^F^+^ population (Fig. 4b,c).

Recently, Han and colleagues have documented high-resolution RNA sequencing analysis on single mid-gastrulation mouse embryos to assemble a spatial transcriptome that correlated with the patterning of cell fates within the embryo^33^. Using the web portal iTranscriptome (www.itranscriptome.org)^33^, we analysed the expression pattern of various genes expressed in the E^+^F^+^ population (Supplementary Fig. 3c). In support of our RNA-Seq data, we found the expression pattern of *Jdp2* and *Epas1* to be co-localized to that of *Eng* as well as the haematopoietic transcription factor *Gata2*, which were observed in the posterior region at this developmental stage. Interestingly, this region was also found to co-express *Bmp2* and *Wnt3*.

Since the earliest haematopoietic precursors express Eng during embryogenesis^9^, we examined Jdp2 expression in E7.5 embryos. Confocal imaging showed expression of Jdp2 at this early developmental stage, with the majority of Jdp2^+^ cells co-expressing GATA1, indicative of primitive erythrocytes (Fig. 4d). Considering that a subpopulation of Eng^+^ cells co-expresses Gata1 in E7.5 YSs^9^, these data further corroborate Jdp2 as a target of Eng and a novel regulator of early haematopoiesis.

Next, we determined whether Jdp2 or Epas1 would have the capacity to rescue the impaired haematopoiesis observed when E^+^F^+^ cells are exposed to IWR-1 (Fig. 3b). For these rescue experiments, E^+^F^+^ cells were transduced with lentiviral constructs encoding Jdp2 or Epas1 and control vector, reaggregated in the presence of IWR-1 for 24 h, and plated for scoring haematopoietic colony activity. Whereas transduction with Epas1 did not rescue blood formation, Jdp2 was able to counteract the inhibitory effects of IWR-1 on the haematopoietic colony activity of E^+^F^+^ cells (Fig. 4e).

Importantly, iSmad1 S210A mutant day 4 EB cultures that exhibit enhanced haematopoietic colony activity (Fig. 2e), express significantly higher levels of *Jdp2* (Fig. 4f), which correlated with *Smad1* expression (Fig. 4g). To further evaluate the effect of Jdp2 in haematopoiesis, we used a haematopoietic reporter transgenic zebrafish, in which DsRed labels haematopoietic cells that are positive for gata1^34^. For this, *Tg*(*gata1:DsRed*) zebrafish embryos at the 1–2-cell stage were injected with *jdp2-GFP* mRNA or with control *venus* mRNA. In accordance with the findings described above (Fig. 4d–f), *jdp2*-injected embryos displayed enhanced haematopoiesis, as demonstrated by the increased levels of DsRed (gata1^+^) when compared with *venus*-injected control embryos at 48 h.p.f. (Supplementary Fig. 3d). Since the lack of Eng during early blood development results in impaired haematopoietic activity^9^,^10^, which is accompanied by decreased Smad1 phosphorylation^8^,^35^, we next examined whether enforced *Jdp2* expression would counteract the defective haematopoietic phenotype of *Eng*-null EBs. Flk-1^+^ cells isolated from day 3 *Eng*^*−/−*^ EBs were transduced with Jdp2-encoded lentiviral vector or empty vector (control), reaggregated for 24 h, and assayed for haematopoietic colony activity. Significantly increased numbers of haematopoietic colonies were observed upon Jdp2 induction (Fig. 4g), revealing that *Jdp2* is an important downstream effector of Eng. This finding was subsequently validated *in vivo* using Eng-deficient E9.5 YSs, which have defective haematopoietic development^9^. Jdp2-encoding and GFP control lentiviral vectors were introduced into Eng-null (KO) and littermate control (CTL) YSs, and haematopoietic activity was enumerated following their direct plating into methylcellulose-based medium containing haematopoietic cytokines. In agreement with the *in vitro* EB data, we observed rescued haematopoiesis upon Jdp2 transduction in Eng-null YS (Supplementary Fig. 3e). Together, these data demonstrate that *Jdp2* is a key target coordinately regulated by Wnt and Eng that is sufficient to establish haematopoietic fate in early mesoderm.

## Discussion

Our study has revealed Eng as a critical modulator of the crosstalk observed between BMP and Wnt signalling pathways during early cell fate specification of the cardiac and haematopoietic lineages. In this context, Eng ensures the commitment of *Bry*^+^ mesodermal precursors (GFP^+^Eng^+^) towards the haematopoietic lineage by repressing the cardiac program in hemogenic sites. Importantly, we have identified *Jdp2* as a target of Eng-mediated BMP and Wnt signalling, providing a molecular mechanism that illustrates how signalling pathways and their targets orchestrate the haematopoietic commitment of mesoderm.

During embryogenesis, BMP signals are not only required for mesoderm induction but also for blood commitment^36^. Moreover, forced expression of Smad1 enhances hemangioblast activity and the haematopoietic commitment of these cells towards the haematopoietic lineage^37^. Similar to BMP, the Wnt signalling pathway plays pivotal roles in development^38^, and specifically in the haematopoietic system, Wnt signals are required for primitive haematopoiesis^39^,^40^. Interestingly, crosstalk between BMP and Wnt has been reported to be involved in the activation of the haematopoietic program in mesodermal cells^5^,^6^. Our findings provide key insights into the mechanism behind this crosstalk, as they reveal that Eng modulates BMP and Wnt signals to enhance Smad1 activity and ultimately haematopoiesis. We further demonstrated that BMP and Wnt integration via Smad1 is critical to regulate gene expression in mesodermal cells. Notably, Smad1 binding was detected at *Tbx5*, suggesting a potential mechanism of Smad1-mediated *Tbx5* repression. Accordingly, reduced levels of Tbx5 have been reported upon Eng induction^7^. Moreover, we revealed that *Jdp2* is regulated by Eng-mediated BMP and Wnt modulation and plays a critical function in promoting the haematopoietic commitment from early mesoderm. *Jdp2*, an AP-1 family transcription factor, has been reported to modulate chromatin by inhibiting histone acetyltransferase activity^41^. Interestingly, *Jdp2* expression was shown to be important for the lymphoid versus myeloid lineage decision by modulating DNA methylation^42^. Neutrophil activity has been reported to be impaired in *Jdp2*^−/−^ mice^43^. A function for *Jdp2* during embryogenesis had not previously been documented. Based on these studies, we speculate that BMP- and Wnt-mediated *Jdp2* induction may be required for directing epigenetic modification of mesodermal progenitors, allowing them to acquire the haematopoietic fate.

Together, our data support the notion that Eng is critical for the crosstalk between BMP and Wnt pathways during development and contribute to broaden our understanding of the transforming growth factor-β superfamily signalling in embryonic cell fate decisions.

## Methods

### Generation of inducible ES cell lines

WT mSmad1 (ref. 44) was subcloned into the p2Lox TEV FLAG targeting vector. p2Lox TEV FLAG vector was generated by inserting the sequence 5′- CTGCAGGCTAGCgcggccGAGAATTTGTATTTTCAGGGTaactacaacatccctaccaccggcggcggcggcggcGACTACAAAGACGATGACGACAAGTAG-3′ flanked by EcoR1 and Not1 sites into the p2lox plasmid. Smad1 S210A mutant was generated using the Quickchange Mutagenesis kit (Stratagene) following the manufacturer's instructions. Smad1 WT and Smad1 S210A inducible ES cells were generated in A2Lox ES cells^7^. To generate 7TFC;iEng ES cells, iEng ES cells were transduced with a lentiviral vector expressing 7xTcf-FFluc//SV40-mCherry (7TFC, Addgene). iEng ES cells containing the 7TFC transgene were obtained following two rounds of FACS sorting based on mCherry expression using a FACS ARIA instrument (BD).

### Growth and differentiation of ES cells

In addition to the generated ES cell lines described above, E14, Bry/GFP-reporter^25^, *Eng*^−/−^10 and iEng ES cells^7^ were used in this study. ES cell lines were cultured on irradiated mouse embryonic fibroblasts in DMEM (Gibco) supplemented with 1,000 U ml^−1^ LIF (Millipore), 15% inactivated fetal bovine serum (Gibco), 0.1 mM non-essential amino acids (Gibco) and 0.1 mM of β-mercaptoethanol (Sigma). For EB differentiation, ES cells were preplated for 30 min to remove mouse embryonic fibroblasts and then plated in hanging drops (100 cells per 10 μl drop) in EB differentiation medium, IMDM (Gibco) supplemented with 15% fetal bovine serum (FBS; Gibco), 4.5 mM monothioglycerol (Sigma), 100 μg ml^−1^ ascorbic acid (Sigma) and 200 μg ml^−1^ iron-saturated transferrin (Sigma) in 150 mm Petri dishes. After 48 h, EBs were collected and resuspended into 10 cm Petri dishes in 10 ml of EB differentiation medium. These dishes were cultured on a slowly swirling table rotator (80 r.p.m.). To induce appropriate Eng expression during EB differentiation, doxycycline (Sigma) was added to the cultures, at 1 μg ml^−1^, beginning at day 2, except for time window studies. In the studies involving iSmad1 ES cell lines, 3 μM of CHIR99021 or dimethylsulphoxide (vehicle) was added to EB cultures at day 3.5 and 24 h later, cells were collected for EryP CFC assay.

### Zebrafish embryology and imaging

WT and transgenic zebrafish lines, *Tg(7xTCF:mCherry)*^19^, *Tg(cmlc2:EGFP)*^45^ and *Tg(gata1:DsRed)*^34^ were maintained according to the IACUC, UMN approved protocols. The full length of Eng CDS (WT) was PCR amplified using human ENDOGLIN cDNA (Dharmacon, MHS1010-202743199). ENDOGLIN deletion mutant (ΔOD)^20^ was generated using the Quickchange Mutagenesis kit (Stratagene) following the manufacturer's instructions. Then, amplified DNA was cloned into pCS2+ vector. The zebrafish jdp2-GFP construct was generated by synthesis of a double-stranded DNA fragment (gBlock, IDT), containing zebrafish *jdp2* CDS without stop codon and flanked by Nhe1 sites. This fragment was subsequently cloned into pCS2-T2A-GFP vector and sequenced. mRNA was synthesized using the mMESSAGE mMACHINE SP6 kit (Ambion) according to the manufacturer's instructions. Zebrafish embryos were injected with 100 pg of control (*venus* or *mCherry*) or human *ENG* mRNAs (WT and ΔOD) at the one–two-cell stage. For confocal live imaging, micro-injected *Tg(cmlc2:EGFP)* embryos were mounted in low-melt agarose, and imaged using an LSM710 confocal microscope (Carl Zeiss Microscopy). For *in vivo* Wnt signalling tracing, micro-injected *Tg(7xTCF:mCherry)* embryos were dissociated in 0.25% trypsin for 3 min at 37 °C and analysed based on mCherry fluorescence by flow cytometry. For counting cardiac progenitors, micro-injected *Tg(cmlc2:EGFP)* embryos were dissociated in 0.25% trypsin for 3 min at 37 °C and analysed based on EGFP fluorescence by flow cytometry. For evaluation of haematopoiesis, 50 ng of control (*venus*) or zebrafish *jdp2-GFP* mRNAs were injected into *Tg(gata1:DsRed)* embryos. mRNA-injected embryos were dissociated in 0.25% trypsin for 5 min at 37 °C and analysed by flow cytometry for DsRed fluorescence at 48 h.p.f. Approximately 20 embryos were grouped as one sample for qPCR with reverse transcription and cell sorting experiments.

### Mice and embryo explants

All animals were handled in strict accordance with good animal practice as defined by the relevant national and/or local animal welfare bodies, and all animal work was approved by the University of Minnesota Institutional Animal Care and Use Committee. Male heterozygous mice bearing an EGFP knock-in into the *Brachyury* locus (GFP/*Bry*^+/−^)^46^ were mated with CD-1 IGS female mice (Charles River Laboratories). *Eng*-deficient embryos were generated by timed mating *Eng*^+/−^ heterozygotes, or Eng^fl/Δ^ to Eng^wt/Δ^, as previously described^9^,^47^. Dox-iEng embryos were generated by timed mating of iEng mice. All YSs were dissociated in 0.25% trypsin for 3 min at 37 °C.

### Generation of iEng transgenic mice

The Dox-iEng ES cell line was generated as previously reported^7^. Mice were derived from the iEng ES cells through blastocyst injection, crossed C57/BL6 to establish a founder bearing the iEng gene and then bred to Rosa-rtTA2SM2 mice^48^ to establish an iEng mouse. Transgenic mice were screened for DNA integration by PCR. To induce Eng expression during gastrulation, 100 μl of 10 mg ml^−1^ doxycycline (Sigma) was given IP to E7.5 time pregnant iEng females.

### Flow cytometry and FACS sorting

Trypsinized EB cells were resuspended in PBS supplemented with 10% FBS containing 0.25 μg per 10^6^ cells of Fc block (eBioscience) and incubated on ice for 5 min. PE-Cy7-conjugated (BioLegend) or PE-conjugated (eBioscience) anti-mouse Eng/CD105 and APC-conjugated anti-mouse Flk-1 (eBioscience) antibodies were added at 0.5 μg per 10^6^ cells and incubated at 4 °C for 20 min before washing with PBS. We analysed stained cells on a FACS Aria after adding propidium iodide (Pharmingen) to exclude dead cells. Day 3 EBs from Bry/GFP ES cells were gated based on GFP expression, and then purified based on the expression of Eng and Flk-1. Individual E8.5 iEng embryo was removed from YS and dissected into head forming region. Both YSs and head forming region of embryos were dissociated in 0.25% trypsin for 3 min at 37 °C. Trypsinized embryos and YSs were analysed by flow cytometry using PE-conjugated anti-mouse PDGFRα, APC-conjugated anti-mouse Flk-1 (eBioscience) and PE-Cy7-conjugated anti-mouse Eng/CD105 (BioLegend) antibodies. Trypsinized zebrafish embryos were stained with PE-conjugated anti-human Eng/CD105 antibody (eBioscience). All antibodies were added at 0.5 μg per 10^6^ cells. Data were analysed using FlowJo Version 7.6.5 software (TreeStar).

### Lentiviral transduction

Cells that had been dissociated from E8.5 GFP/*Bry*^+/−^ embryo explants were transduced either with empty vector control (pSAM-iresGFP) or a lentiviral vector encoding Eng (pSAM-Eng), reaggregated in 1 ml EB medium on low-adherent 24-well plates. After 24–48 h, reaggregates were used for cardiac differentiation studies, as described below. For differentiating ES cells, the E^+^F^+^ subfraction purified from day 3 Bry/GFP EBs were transduced either with lentiviral vector encoding Jdp2 (pRRL-Jdp2) or Epas1 (pRRL-Epas1). As a control, a cohort was transduced with empty vector (pRRL-dsRed). For E9.5 YSs, dissociated YSs were divided in two and transduced either with lentiviral vector encoding Jdp2 (pRRL-Jdp2) or empty vector (pRRL-GFP). Spin-infections were performed at 1,100*g* for 1 h and 30 min at 30 °C and then incubated in the presence of the lentivirus for an additional 24 h at 37 °C.

### Haematopoietic CFC assays

Cells from day 4 EBs or reaggregates were plated at 5 × 10^4^ cells into 1.5 ml of methylcellulose medium containing interleukin-3, interleukin-6, erythropoietin (EPO) and SCF (M3434; StemCell Technologies). In the studies involving YS, 10,000 cells from E8.5 iEng YSs or E9.5 Eng-floxed YSs were plated directly into M3434 and cultured in a humidified low-oxygen (5%) incubator at 37 °C. Primitive erythroid colonies were counted after 5 days, and definitive haematopoietic colonies were scored 7–10 days after plating.

### Cardiac differentiation

Reaggregates from EB-sorted cells were plated on gelatinized plates in IMDM medium with 2% FBS and 10 μM IWR-1 (Sigma). Reaggregates from E8.5 embryo explants were plated on OP9 monolayer in the absence or presence of 10 μM IWR-1.

### Immunofluorescence staining

Cells for immunofluorescence staining were fixed with 4% paraformaldehyde in PBS, and blocked with 5% bovine serum albumin (BSA) in PBS. cTnI (1:250, Abcam) and anti-Goat Cy3 (1:500, Jackson Laboratory) were used as primary and secondary antibodies, respectively. To detect the nuclei, after washing, samples were stained with HOECHST H1399 (1:2,000, Life Technologies). For quantification, the size of cTnI-positive (cTnI^+^) cells from embryo explants were measured by ImageJ (NIH). For YS analysis, dissected embryos were fixed in 2% paraformaldehyde for 20 min, subsequently incubated in 5 and 15% sucrose solutions until embryos decanted and then frozen in 7.5% gelatin. Cryosections were incubated with anti-Jdp2 (1:100, Santa Cruz) and anti-Gata1 (1:100, Santa Cruz) antibodies overnight at 4 °C. Reactions were detected after 1 h of incubation with anti-Rabbit Alexa Fluor 555 (1:400, Invitrogen) and anti-Rat 488 (1:400, Jackson Laboratory). Staining was visualized by laser confocal scanning (Zeiss LM510).

### Western blotting

EB cell lysates were prepared using 1 × RIPA buffer (150 mM NaCl, 50 mM Tris-HCl, pH 7.5, 1 mM EDTA, 1% Triton, 1% sodium deoxycholate and 0.1% SDS) supplemented with Complete Protease Inhibitor Cocktail (Roche) and PhosSTOP (Roche), and quantified with Bradford reagent (Sigma). Samples were prepared in Laemmli buffer (BioRad) and loaded on gels for SDS–polyacrylamide gel electrophoresis. Proteins were transferred to a polyvinyl difluoride membrane (Millipore). The following primary antibodies were applied at the indicated dilution in Primary Antibody Signal Boost Immunoreaction Enhancer (Calbiochem); phosphorylated Smad1/5/8 (S465/8 and S426/8, 1:1,000, Cell Signaling), Smad1 (1:3,000, Abcam), cTnI (1:1,000, Abcam), β-active Catenin (1:1,000, Millipore), pGsk3α/β (S21/S9, 1:1,000, Cell Signaling). Gapdh (Abcam) and actin (Millipore) were diluted at 1:3,000 with 5% BSA in 1 × TBS–Tween 20. All primary antibodies were incubated for 12 h at 4 °C on a shaker. Enhanced chemiluminescence peroxidase-labelled anti-mouse and anti-rabbit antibodies (GE Biosciences) were diluted at 1:20,000 with 5% BSA in 1 × TBS–Tween 20. After washing the membranes, SuperSignal West Pico Chemiluminescent Substrate (ThermoScientific) was utilized to detect the horseradish peroxidase signal. All uncropped western blots can be found in Supplementary Fig. 4.

### Reporter assay

Wnt activity was measured in iEng;7TFC reporter ES cells, in which fire fly luciferase reflects β-catenin expression (7xTcf-FFluc//SV40-mCherry; Addgene). To stimulate the Wnt pathway, 3 μM CHIR99021 (Millipore) was added to the culture medium at day 3 of EB differentiation. EBs were collected at 30 min, 1 and 3 h after Wnt stimulation. A control sample was collected before Wnt stimulation, which we consider 0 h sample. To measure bioluminescence in these samples, we utilized the Dual-luciferase reporter assay system (Promega), and luciferase activity was analysed using the Sirus Luminometer (Berthold Detection Systems).

### RNA isolation, gene expression and RNA-Seq

Cells from explants, YSs, zebrafish embryos and total EBs were resuspended in Trizol (Life Technologies) and processed following the manufacturer's instructions. RNAs from sorted cells were retro-transcribed using Superscript Vilo (Life Technologies) and the rest RNAs were retro-transcribed using Thermoscript (Life Technologies). TaqMan probes (Life Technologies) were used. For globins, we designed customized primer/probe sets (all shown 5′–3′): beta-major F, AGGGCACCTTTG CCAGC; beta-major R, GGCAGCCTCTGCAGCG; beta-major probe, 6FAM-CGTGATTG TGCTGGGCCACCACCT-TAMRA. Embryonic F, CCTCAAGGAGACCTTTGCTCAT; embryonic R, CAGGCAGCCTGCACCTCT; embryonic probe, 6FAM-CAACATGTTGG TGATTGTCCTTTCT-TAMRA.

For RNA-Seq, 100 ng of total RNA for each sample was treated with DNase1 amplification grade (Life Technologies) following the manufacturer's instructions. Sequencing libraries were generated from 100 ng of total RNA using the TruSeq RNA Sample Preparation kit (Illumina) and quantitated using the Qubit fluorometer (Life Technologies) following the manufacturer's instructions. The libraries were then pooled six samples per lane using 35 ng per sample for a 51+8+8 cycle (to accommodate 8+8 indexed samples in other lanes) Single Read run on the HiSeq 2500 (Illumina) by high-output run sequencing. The sequencer outputs were processed using Illumina's CASAVA-1.8.2 basecalling software. Demultiplexing assigned ∼475 million reads across the 18 samples, ranging from 23 to 30 million reads per sample. Of the assigned reads, ∼2.5 million were discarded for low quality or the presence of sequencing adaptors in the reads. Each sample's reads were then processed using RSEM version 1.2.3 (with bowtie-0.12.9 for the alignment step)^49^,^50^. Percentage of reads mapped to the transcriptome ranged from 88 to 93%.

### ChIP, library generation, sequencing and data analysis

ChIP was performed following the protocol described by Young and colleagues^51^ with minor modifications. In brief, D4 EBs were trypsin-treated at 37 °C for 1 min with gentle shacking and reaction was inhibited by adding 10%FBS/PBS. Single cells were washed once with PBS, resuspended in 10%FBS/PBS and supplemented with formaldehyde (final concentration 1%) for crosslinking of protein–DNA complexes (10 min at room temperature) followed by quenching with glycine. Cells were snap-frozen in liquid nitrogen and stored at −80 °C if not processed immediately. Cell pellets were incubated in lysis buffer LB1 supplemented with protease inhibitors (50 mM HEPES KOH, pH7.5, 140 mM NaCl, 1 mM EDTA, 10% glycerol, 0.5% NP40, 0.25% Triton X-100+Complete-mini—Roche) for 10 min at +4 °C followed by incubation in buffer LB2 supplemented with protease inhibitors (10 mM Tris-HCl, pH 8, 200 mM NaCl, 1 mM EDTA, 0.5 mM EGTA+Complete-mini—Roche) for 10 min at +4 °C. Cell pellet was then resuspended in LB3 supplemented with protease inhibitors (10 mM Tris–HCl, pH 8, 100 mM NaCl, 1 mM EDTA, 0.5 mM EGTA, 0.1% sodium deoxycholate, 0.5% *N*-lauroylsarcosine+Complete-mini—Roche) and then sonicated with a Branson sonicator at 18% power for 1 min with intervals of 10 s ON–10 s OFF. Each sample was subjected to five to six cycles of sonication to reach an average chromatin size of 300 bp. After shearing, samples were centrifuged for 10 min at 16,000*g* and snap-frozen in liquid nitrogen if not processed immediately. For each ChIP, 20 μg of chromatin (diluted to 400 μl) was precleared for 4 h at 4 °C with 15 μl of BSA-blocked Protein A-conjugated sepharose beads (GE healthcare) and then supplemented with 1/10 volume of 10% Triton X-100. Immunoprecipitation was performed by overnight incubation with normal rabbit IgG (8 μg—Santa Cruz Biotechnology) or anti-Smad1 antibody (1:25—Cell Signaling Technology #9743). Immune complexes were recovered by incubation with 15 μl of BSA-blocked Protein A-conjugated sepharose beads for 4 h at 4 °C and then washed five times with RIPA wash buffer (50 mM HEPES KOH, pH7.5, 500 mM LiCl, 1 mM EDTA, 1% NP40, 0.25% Triton X-100 and 0.7% sodium deoxycholate) and one time with TEN buffer (10 mM Tris-HCl, pH 8, 1 mM EDTA and 50 mM NaCl). Immunoprecipitated chromatin was recovered by incubating beads with 200 μl of elution buffer (50 mM Tris–HCl, pH 8, 10 mM EDTA and 1% SDS) for 20 min at 65 °C. Chromatin from immunoprecipitation and input (equivalent to 1% of starting material) was reverse crosslinked overnight at 65 °C, then diluted 1:1 with TE (10 mM Tris–HCl, pH 8, and 1 mM EDTA) supplemented with 4 μl of RNaseA 20 mg ml^−1^ and incubated for 2 h at 37 °C followed by Proteinase K treatment (4 μl of 20 mg ml^−1^ stock for each sample) for 30 min at 55 °C. DNA was purified by Phenol–chloroform–isoamyl alcol extraction (twice) followed by chloroform extraction, then supplemented with 1/10 of volume of 3 M sodium acetate, pH 5, and 1.5 μl of glycogen and precipitated with two volumes of 100% ethanol at −80 °C for >1 h. Followed 30 min centrifuge at 16,000*g*, pellet was washed with 75% ethanol, air-dried and dissolved in 45 μl H_2_O. qPCR was performed in a final volume of 10 μl using SYBR Premix Ex Taq II (Clontech), 0.5 μl of 1.4 μM primer stock and 0.3 μl sample/reaction, and run on a 384-well plate on a ABI7900HT instrument (Applied Biosystems). The following primers were used for qPCR. Id1 −1 Kb-FW: 5′-AGCCCGTCCGGGTTTTATG-3′; Id1 −1 Kb-RV: 5′-GTGTCAGCGTCTGAACAAGC-3′; Id2 −2 Kb-FW: 5′-CCCTGCAGCCTTGTCCTC-3′; Id2 −2 Kb-RV: 5′-CCTTGCAGGCATTGATCAGC-3′; Tbx5 +6 Kb-FW: 5′-TGGATCTCACGGAGGATGGA-3′; Tbx5 +6 Kb-RV: 5′-GGGCGAGATTCGTGCAGATA-3′.

Libraries were generated following a gel-free protocol using AMPure XP beads (Beckman Coulter) for all the purification and size selection steps. An amount of 10 ng or less of DNA was end-repaired using End-it DNA end repair (Epicentre), then A-tailed using Klenow Fragment (3′→5′ exo^−^, NEB) followed by adapter-barcode ligation using T4 DNA ligase (Enzymatics). Illumina compatible adapter-barcodes were purchased from BIOO scientific. After ligation, DNAs were negatively size selected using 0.5 × Ampure XP beads and unbound DNAs were positively size selected by adding 0.4 × Ampure XP beads (this step allows for retention of DNA fragments ranging 200–500 bp). Libraries were amplified using Phusion High Fidelity PCR master mix 2 × (NEB) with a 16-cycle program. Purified libraries were then submitted to the University of Minnesota Genomic Center (UMGC) for quantification, quality control and sequencing. Libraries were pooled and sequenced on a lane of Single-End run on a HiSeq2500 operated at High-Output mode (Illumina). The sequencer outputs were processed using the Galaxy platform^52^ available at the Minnesota Supercomputing Institute. Demultiplexing assigned ∼134 million reads across the four samples, ranging from 30 to 36 million reads per sample. Each sample's reads were then aligned to the mouse genome (mm10) using Bowtie2 (ref. 53) followed by the removal of PCR duplicates using the SAM tool rmdup 1.0.1 (ref. 54). Peak calling was performed using both MACS 1.0.1 (ref. 55) (parameters: -s 51 -bw 300, -p 1e-05 -m 32) and QESEQ^56^ (parameters: -s 100 -c 15 -p 0.05) and only common peaks between the outputs of both algorithms were further considered. Biwwig files for visualization on IGV^57^ were generated by converting the wig files obtained from MACS using the Galaxy tool Wig-to-BigWig. Density maps were generated with SeqMiner^58^ using the enrichment file enr.sgr produced by QESEQ.

### Statistical analysis

Differences between two groups were assessed by using the Student's *t*-test. Differences between multiple groups were assessed by analysis of variance. The R package EBSeq^59^ was used to identify genes expressed differentially between one sample type and one or more others. Those genes reported by EBSeq to have a posterior probability of differential expression of 0.50 or greater were then filtered to select only those with median-normalized TPM >16 in at least one sample, and a fold change of at least 2 between the mean of the median-normalized expected counts (EC) of the control samples versus that of the others. Heatmaps of relative expression display for each gene G and sample S the value log_2_ ((1+median-normalized EC(G,S))/(1+mean overall S_i_ median-normalized EC(G, S_i_)).

### Data availability

RNA-Seq data that support the findings of this study have been deposited in the Gene Expression Omnibus (GEO) database under accession code GSE85855. ChIP-seq data have been deposited in the GEO database under accession code GSE85797. All other relevant data are available from the corresponding author on request.

## Additional information

**How to cite this article:** Baik, J. *et al*. Endoglin integrates BMP and Wnt signalling to induce haematopoiesis through JDP2. *Nat. Commun.* **7,** 13101 doi: 10.1038/ncomms13101 (2016).

## Supplementary Material

Supplementary Information Supplementary Figures 1-4

Supplementary Movie 1 Contractile cardiomyocytes are observed only in the E^+^ F^-^ sub-fraction. Reaggregates from unsorted, E^+^ F^-^ and EF^-^ sub-fractions were plated as monolayer onto OP9 stromal cells for 5 days in the presence of IWR-1 or DMSO. Only IWR-1-treated E^+^ F^-^ cultures generated spontaneously beating cardiomyocytes (a). Reaggregates from unsorted cells (b) and EF^-^ cells (c) do not generate beating cardiomyocytes.

Supplementary Movie 2 Contractile cardiomyocytes are observed only in the E^+^ F^-^ sub-fraction. Reaggregates from unsorted, E^+^ F^-^ and EF^-^ sub-fractions were plated as monolayer onto OP9 stromal cells for 5 days in the presence of IWR-1 or DMSO. Only IWR-1-treated E^+^ F^-^ cultures generated spontaneously beating cardiomyocytes (a). Reaggregates from unsorted cells (b) and EF^-^ cells (c) do not generate beating cardiomyocytes.

Supplementary Movie 3 Contractile cardiomyocytes are observed only in the E^+^ F^-^ sub-fraction. Reaggregates from unsorted, E^+^ F^-^ and EF^-^ sub-fractions were plated as monolayer onto OP9 stromal cells for 5 days in the presence of IWR-1 or DMSO. Only IWR-1-treated E^+^ F^-^ cultures generated spontaneously beating cardiomyocytes (a). Reaggregates from unsorted cells (b) and EF^-^ cells (c) do not generate beating cardiomyocytes.
