# Supplementary material for: Endoglin integrates BMP and Wnt signalling to induce haematopoiesis through JDP2
Source: Nat Commun. 2016 Oct 7;7:13101. doi: 10.1038/ncomms13101 (PMC5059784; doi:10.1038/ncomms13101)
Supplement: Supplementary Information — Supplementary Figures 1-4 [file ncomms13101-s1.pdf]

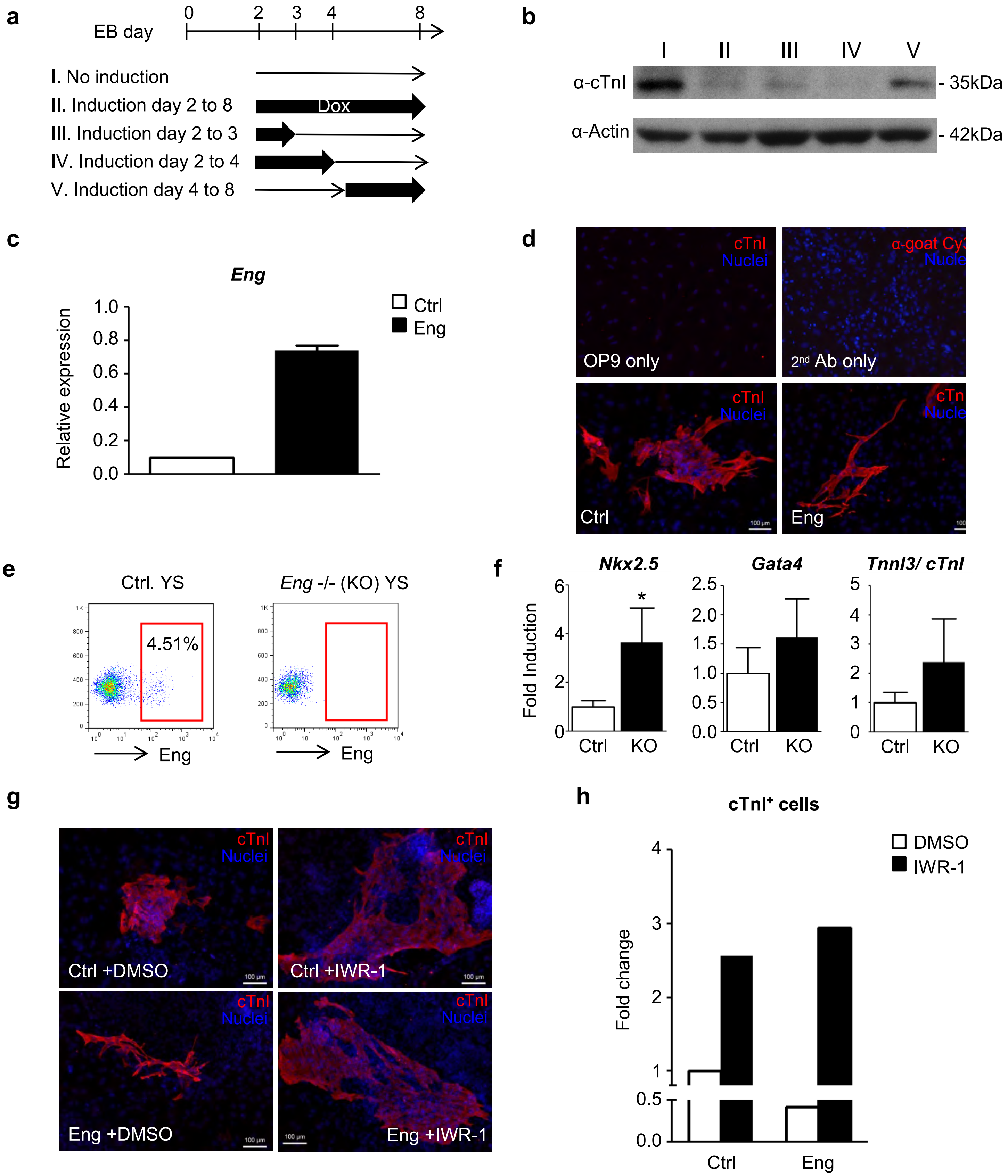
a
EB day
0 2 3 4 8
I. No induction
II. Induction day 2 to 8
Dox
III. Induction day 2 to 3
IV. Induction day 2 to 4
V. Induction day 4 to 8
b
I II III IV V
α-cTnI
- 35kDa
α-Actin
- 42kDa
c
Eng
Relative expression
1.0 0.8 0.6 0.4 0.2 0.0
Ctrl
Eng
d
cTnI
Nuclei
OP9 only
2nd Ab only
Ctrl
Eng
e
Ctrl. YS
Eng -/- (KO) YS
4.51%
Eng
f
Nkx2.5
Gata4
Tnnl3/ cTnI
Fold Induction
Ctrl KO
g
Ctrl +DMSO
Ctrl +IWR-1
Eng +DMSO
Eng +IWR-1
h
cTnI+ cells
DMSO
IWR-1
Fold change
Ctrl
Eng

Supplementary Figure 1. Effect of endoglin induction on cardiac differentiation.

(a) Outline of time windows of endoglin induction during EB differentiation (D2-D8). The following 5 experimental groups were investigated: I) no dox added to cultures, II) dox added for the whole period from D2-D8 of EB differentiation, III) dox added for 24 hours from D2-D3, IV) dox added for 48 hours from D2-D4, and V) dox added for 96 hours from D4-D8. (b) Western blot analyses for cTNI in day 8 EB cultures reveal that early endoglin induction (II, III, IV) suppresses cardiac differentiation. α- Actin was used as loading control. (c-d) Effect of endoglin induction in embryo explants. (c) qPCR analyses confirm up-regulation of Endoglin in E8.5 mouse explants that had been transduced with Eng lentivirus (pSAM-Eng) when compared to control cultures transduced with empty vector (pSAM). (d) Immunostaining for cTNI reveals the presence of several and large clusters of cardiac cells in control (Ctrl) explants cultured onto OP9 stromal cells (lower left), whereas endoglin-induced explants displayed smaller and fewer cTnT+ clusters (lower right). cTnI is shown in red and nuclei in blue. Scale bars: 100µm (e-f) Expression of cardiac genes in the YS of Eng-/- embryos. (e) FACS analyses confirm the null phenotype (right panel). (f) Up-regulation of Nkx2.5 in the yolk sacs of Eng-/- mice. Bars indicate standard errors from 2 independent experiments. Eng-/- YS (n=3) and control YS (n=5). *$p<0.05$ by *t*-test. (g and h) Rescued cardiogenesis when Eng-induced embryo explants are treated with IWR1. (g) Representative immunostaining for cTnI. Control cultures were treated with DMSO (left panels). Scale bars: 100µm. (h) Quantification of cTnI staining (g) confirms rescue of cardiac suppression when endoglin-induced explants are treated with IWR-1. Results are shown as fold-change of cTnI+ cells.

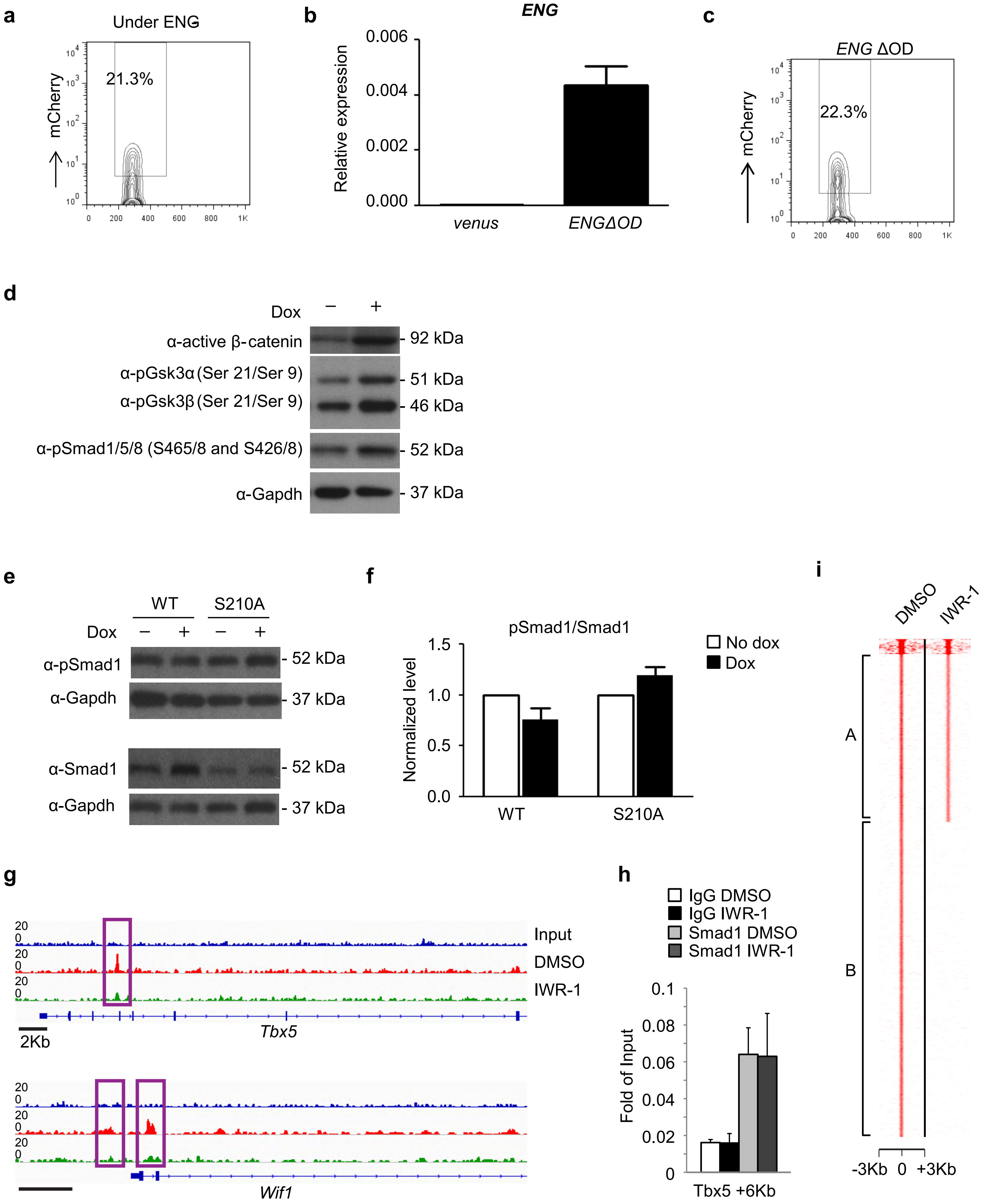

a
Under ENG
mCherry
21.3%
b
ENG
Relative expression
0.006
0.004
0.002
0.000
venus
ENGΔOD
c
ENG ΔOD
mCherry
22.3%
d
Dox
−
+
α-active β-catenin
92 kDa
α-pGsk3α (Ser 21/Ser 9)
51 kDa
α-pGsk3β (Ser 21/Ser 9)
46 kDa
α-pSmad1/5/8 (S465/8 and S426/8)
52 kDa
α-Gapdh
37 kDa
e
WT
S210A
Dox
α-pSmad1
52 kDa
α-Gapdh
37 kDa
α-Smad1
52 kDa
α-Gapdh
37 kDa
f
pSmad1/Smad1
Normalized level
No dox
Dox
WT
S210A
g
Input
DMSO
IWR-1
Tbx5
2Kb
Wif1
h
IgG DMSO
IgG IWR-1
Smad1 DMSO
Smad1 IWR-1
Fold of Input
Tbx5 +6Kb
i
DMSO
IWR-1
A
B
-3Kb
0
+3Kb

Supplementary Figure 2. Eng modulates integration of BMP and Wnt signaling in mesodermal cells.

Full length ENDOGLIN (ENG) and control venus mRNAs were injected into Tg(7xTCF:mCherry) zebrafish embryos. (a) mCherry fluorescence gated in the ENG-negative cell fraction of ENG- injected embryos. (b-c) Injection of ENG mutant into Tg(7xTCF:mCherry) zebrafish embryos. ENG ΔOD mRNA injection was confirmed using ENG N' specific probes. (c) FACS analysis shows that mCherry levels in these embryos are similar to controls, around 22% (lower panel). (d) Western blot confirms enhanced levels of pSmad1/5/8 and active β-Catenin in D4 Eng- induced EBs, which are accompanied by increased phosphorylation of the inhibitory forms of GSK3 (pGSK3 α/β S21/9). Gapdh is used as loading control. (e-f) Generation of doxycycline-inducible Smad1 ES cell lines. ES cells were engineered to inducibly express wild-type (WT) or mutant S210A (S210A) Smad1 upon dox treatment. (e) Western blot analysis for Smad1 and phosphorylated Smad1 (pSmad1) in induced and non-induced cultures, and respective quantification (f) following normalization to Gapdh levels. Results were plotted as ratio between phosphorylated Smad1 and total Smad1. Error bars indicate standard errors from three independent experiments. (g) Integrative Genome Viewer (IGV) tracks for Tbx5 and Wif1 genomic regions from Smad1 ChIP-seq experiment performed on D4 EBs treated for 24h with DMSO or IWR-1. Squares indicate Smad1 bound regions. Scale bar: 2Kb. (h) Validation of Smad1 binding at the Tbx5 (+6Kb) site by ChIP-qPCR. Graph represents the average plus standard error from 6 independent experiments. (i) Tag density distribution of ChIP-seq reads across a ±3Kb region centered on Smad1 peaks. IWR-1 treatment affects the genome wide binding of Smad1 in both group A and B.

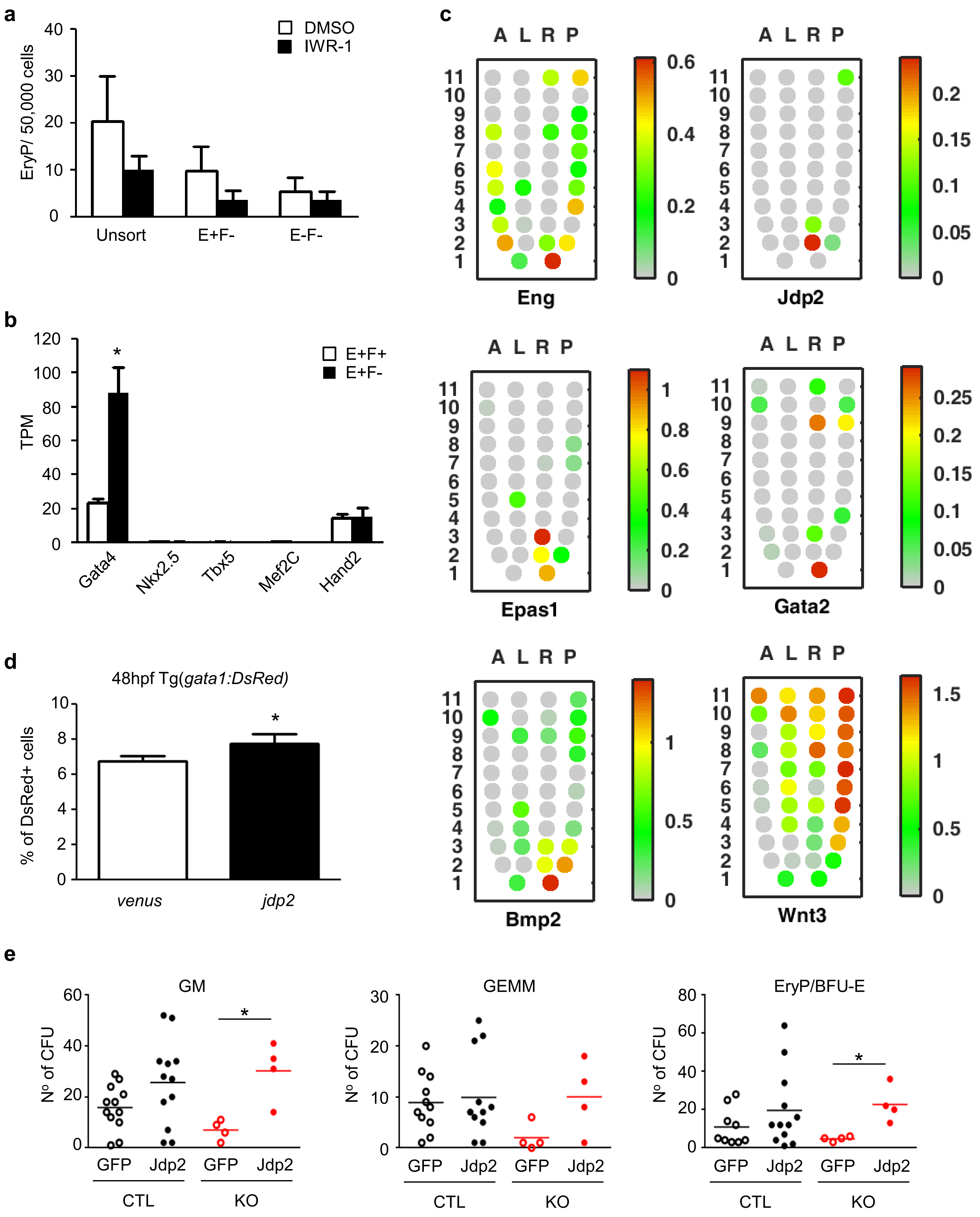

a
EryP/ 50,000 cells
DMSO
IWR-1
Unsort
E+F-
E-F-
b
TPM
E+F+
E+F-
Gata4
Nkx2.5
Tbx5
Mef2C
Hand2
c
A L R P
Eng
Jdp2
Epas1
Gata2
Bmp2
Wnt3
d
48hpf Tg(gata1:DsRed)
% of DsRed+ cells
venus
jdp2
e
GM
GEMM
EryP/BFU-E
Nº of CFU
GFP
Jdp2
CTL
KO

Supplementary Figure 3. Further characterization of GFP+(Bry+) sub-fractions.
(a) Quantification of EryP colonies in D4 GFP+(Bry+)Flk-1- EBs expressing Eng or not (E+F- and E-F-) as well as in the unfractionated population (unsorted). Bars indicate standard errors from 3 independent experiments. (b) Expression levels of Gata4, Nkx2.5, Tbx5, Mef2C, and Hand2 in E+F+, and E+F- sub-fractions are shown as median-normalized transcripts per million (TPM). *$p<0.05$ by *t*-test. (c) The pattern of Eng, Jdp2, Epas1, Gata2, BMP2, and Wnt3 at mid gastrulation stage mouse embryos reveals co-expression of these genes in the distal posterior region. Corn plots were generated using iTranscriptome. (d) Bar graphs show the percentage of DsRed+ (Gata1+) cells, quantified by FACS in jdp2- and control venus-injected embryos, the former showing enhanced hematopoiesis upon jdp2 induction. Bars represent SEM from 3 independent experiments. *$p<0.05$ by *t*-test. (e) Forced expression of Jdp2 rescues impaired hematopoietic colony activity of E9.5 Eng-null YSs. Horizontal bar represents the mean of 2 independent biological replicates performed in duplicate. *$p<0.05$ by *t*-test.

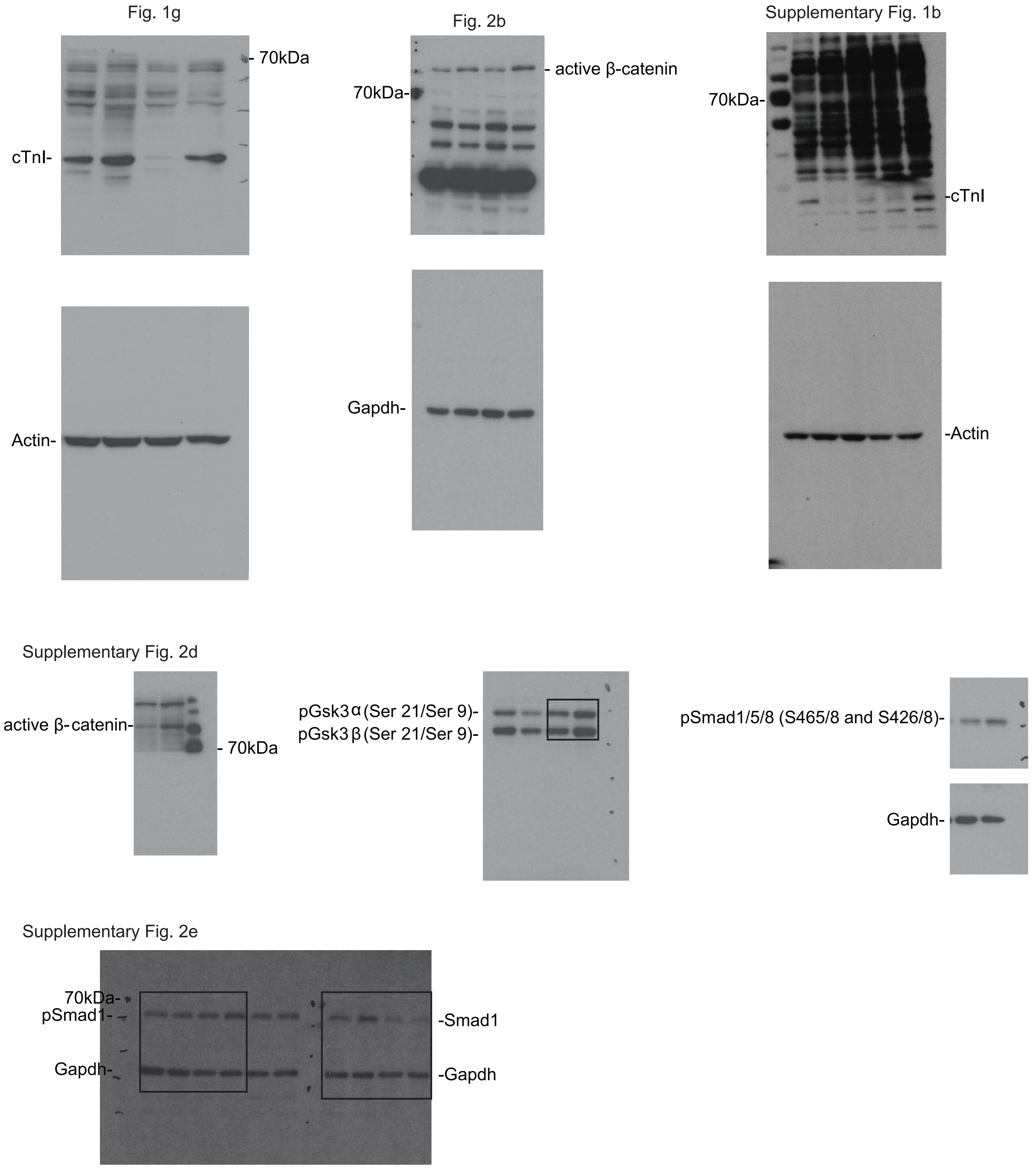

Supplementary Figure 4. Representative original immuno blots.
The cropped area used in the figures are marked by boxes.
